# Heterologous Complementation of SPO11-1 and -2 Depends on the Splicing Pattern

**DOI:** 10.3390/ijms22179346

**Published:** 2021-08-28

**Authors:** Thorben Sprink, Frank Hartung

**Affiliations:** Julius Kuehn-Institute (JKI)—Federal Research Centre for Cultivated Plants, Institute for Biosafety in Plant Biotechnology, 06484 Quedlinburg, Germany; Frank.Hartung@julius-kuehn.de

**Keywords:** meiosis, SPO11, double strand breaks, DSB, plant, splicing, immunostaining

## Abstract

In the past, major findings in meiosis have been achieved, but questions towards the global understanding of meiosis remain concealed. In plants, one of these questions covers the need for two diverse meiotic active SPO11 proteins. In *Arabidopsis* and other plants, both meiotic SPO11 are indispensable in a functional form for double strand break induction during meiotic prophase I. This stands in contrast to mammals and fungi, where a single SPO11 is present and sufficient. We aimed to investigate the specific function and evolution of both meiotic SPO11 paralogs in land plants. By performing immunostaining of both SPO11-1 and -2, an investigation of the spatiotemporal localization of each SPO11 during meiosis was achieved. We further exchanged SPO11-1 and -2 in *Arabidopsis* and could show a species-specific function of the respective SPO11. By additional changes of regions between SPO11-1 and -2, a sequence-specific function for both the SPO11 proteins was revealed. Furthermore, the previous findings about the aberrant splicing of each SPO11 were refined by narrowing them down to a specific developmental phase. These findings let us suggest that the function of both SPO11 paralogs is highly sequence specific and that the orthologs are species specific.

## 1. Introduction

**Figure 1:**
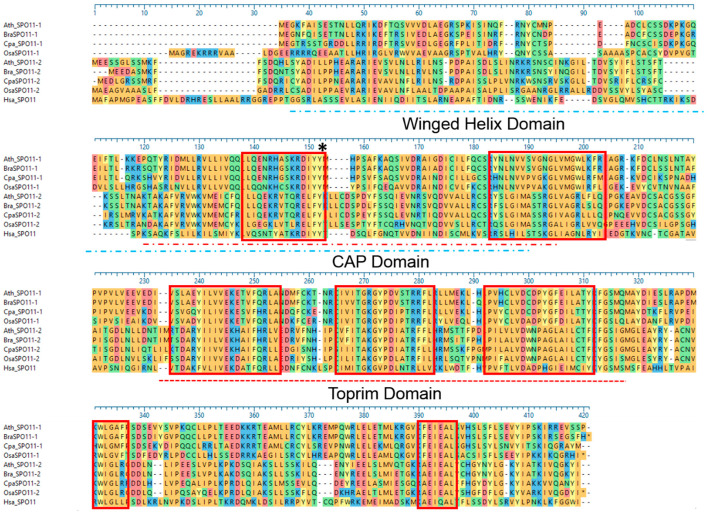
Clustal Ω alignment of SPO11 proteins from different species. Conserved domains are marked with a red box. Active tyrosine is marked with an asterisk. Main domains are named and marked as dashed lines. Ath = *Arabidopsis thaliana*; Bra = *Brassica rapa*; Cpa = *Carica papaya*; Osa = *Oryza sativa*; Hsa = *Homo sapiens*.

Evolution and genetic diversity rely on meiosis, the novel combination of genetic material, achieved mainly by double strand break (DSB) induction, followed by one of the cell’s intrinsic DNA repair mechanisms [1]. In addition, plant breeding uses meiotic DSB-induction to achieve new genetic diversity by crossing, e.g., to improve yield or tackle future challenges such as a changing climate. Lately, additional DSBs are often induced by site-directed nucleases such as CRISPR-Cas9 to break the DNA at a desired locus and induce site directed mutagenesis for breeding purposes but achieving translocations and creating real diversity using SDN is still a very rare event [2]. However, DSBs mainly occur naturally during mitotic or meiotic cell divisions [3]. Especially in meiosis, DSB-induction is the crucial step that ensures overall genome stability by the correct pairing of the homologous chromosomes via a physical linkage on one hand. On the other hand, genetic diversity is enhanced via the resolution of crossovers by the exchange of genetic material between the chromosomes in the developing gametes [4,5,6,7,8,9]. The initiation of crossovers lies in the formation of DSBs by SPO11 at the leptotene stage of early prophase I [1]. SPO11 shows homology to the Topoisomerase VI subunit A (TOPVIA) from archaea [10,11,12,13]. Both proteins share seven conserved motifs, a CAP (cysteine-rich secretory proteins, antigen 5 and pathogenesis-related 1 proteins) domain including a winged helix domain and a TOPRIM (topoisomerase-primase) domain (Figure 1) and are able to cleave double stranded DNA, constituting a persisting 5’-phosphotyrosyl linkage [10,11,13].

**Figure 2:**
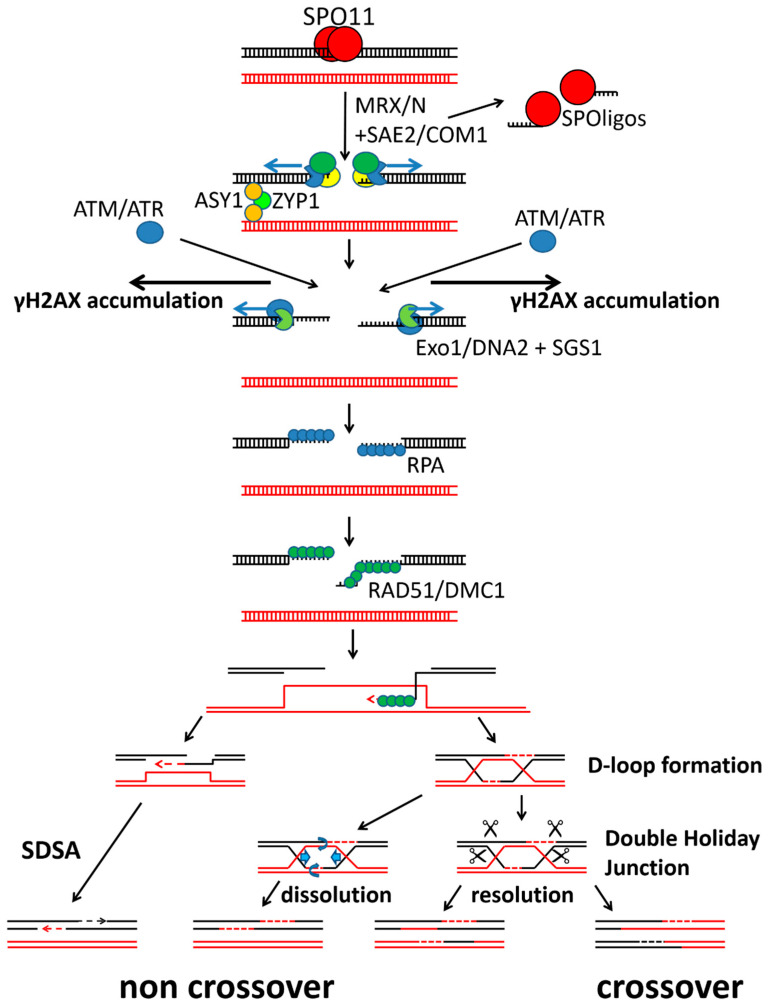
Double strand break (DSB) induction and repair during meiosis. Several key proteins of DSB induction and repair are shown at the stage at which they are active, according to the known processes in model organisms. Proteins that are commonly used for inducing DSBs in several organisms are written in bold. ds DNA = double stranded DNA; SDSA = synthesis-dependent strand annealing; DSB = double strand break.

The release of SPO11 is performed by a combined action of several proteins such as the MRX/N-complex (MRE11, RAD50, XRS2 in yeast or NBS1 in other organism) in combination with SAE2/COM1 [14,15,16,17,18,19,20,21,22,23,24]. Following the removal of SPO11 from the break sites by endonucleolytic cleavage, different specialized meiotic DNA repair proteins including RPA1, RAD51 and DMC1 mediate strand invasion. Several other DNA repair factors perform DNA elongation and the capture of the second DNA strand followed by the subsequent repair and ligation of the break (Figure 2); reviewed in [25,26].

In many organisms, such as mammals and fungi, a single SPO11 is present and sufficient for meiotic DSB formation. In mice and humans, two distinct splice variants of SPO11 have been identified that possess different features concerning the timing of the DSB induction, as studies in mice have shown [27,28]. It is proposed that SPO11 forms multimers or dimers between itself and/or between distinct spliced variants [9,13,29]. The differential splicing of SPO11 is common in mice and humans and is conserved in plants even down to mosses [30,31,32,33] (this study). The process of differential splicing seems to be a common feature in meiosis. It was also identified for other meiosis-specific proteins, such as DMC1 and MER2, in various species including yeast, mammals and plants, pointing towards a conserved mechanism [34,35,36]. Other than mammals, plants encode for at least three different SPO11 proteins, from which two play a meiotic role. In *A. thaliana*, referred to as AthSPO11-1 and AthSPO11-2, both are essential in a functional form for DSB formation during meiosis [11,12,37,38]. The third one, AthSPO11-3, possesses pivotal functions during the somatic development of plant cells in combination with the second subunit of the topoisomerase VI (TOPVIB), but it has no function in meiosis [7,37,38,39]. In rice (*Oryza sativa*), two additional SPO11 proteins have been identified, from which one, OsaSPO11-4, is proposed to also have a function in meiosis [40,41]. The phenotypic behavior of the SPO11-1 and -2 mutants in *Arabidopsis* support the theory that SPO11 in plants acts similarly to its orthologues in archaea and mammals and forms complexes or multimers between each other, which then interact with other factors and/or the DNA to form DSBs [7,9,13]. Studies let us assume that a heterodimer is formed between SPO11-1 and SPO11-2 catalyzed by MTOPIVB, as at least two hybrid assays have shown a complex formation between the N-terminal part of SPO11-1 and -2 and the C-terminal part of MTOPVIB [42]. 

This TOPVIB-like protein named meiotic TOPVIB-like (MTOPVIB-L) has been identified in mice and *Arabidopsis* and is involved in the DSB induction during meiosis, as knockout alleles of these genes are phenotypically identical to *spo11* mutants [42,43,44]. These findings present a protein–protein interaction between AthMTOPVIB and AthSPO11-1 as well as AthSPO11-2. This interplay is unconditionally necessary to form heterodimers between SPO11-1 and -2 in *Arabidopsis*, which is in line with observations that archaeal TOPOVI forms a functional heterotetramer between two A and two B subunits [45]. Vrielynck et al. observed that AthMTOPVIB is located at the chromosomes during early prophase; at the same time, AthSPO11-1 can be visualized using immunostaining at the chromosomes [46] (this study). Taken together, this supports the theory of a multimer- or even a DSB-inducing complex-formation. Protein interaction was, for a long time, only known for AthSPO11-1 with AthPRD1 and for AthSPO11-2 and -3 with AthTOPVIB in vitro [38,47]. In addition, interaction between MTOPVIB with SPO11-2 and PRD3 and DFO was shown in *Arabidopsis* [48]. These data support the assumption of a larger DSB-inducing complex involving both SPO11-1 and -2 during meiosis but no distinct evidence for this hypothesis was found, since no interaction between SPO11-1 and SPO11-2 could be shown in plants. Even though decent progress has been made in understanding the DSB formation in plants, it is still unclear how SPO11-1 and -2 collaborate in meiosis and which regions of the proteins are defining their specific functions. We and others were able to reveal the evolution of SPO11 in all the kingdoms of life and could identify a widely conserved mechanism of differential splicing for both *SPO11-1* and *-2* in numerous plants [13,32,33,49]. Recent findings let us assume that differential splicing is also leading to additional functional forms in plants, as recently shown for SPO11-2 in the A-subgenome of wheat [50].

In this study, we address several questions concerning the function of the two meiotic SPO11 proteins in plants. For this purpose, we evaluated the localization of AthSPO11-1 and -2 during meiosis, by generating for the first time a specific antibody against AthSPO11-2 and used this in a combined immunolocalization with an AthSPO11-1 antibody [46]. Furthermore, we investigated if the function of orthologous *SPO11* genes is conserved between different related plants. By using both genomic DNA and complementary DNA (cDNA) for complementation approaches, we were able to survey if aberrant splicing has any effect on the complementation efficiency. In a second approach, we investigated which regions of SPO11-1 and -2 are defining the different functions of both proteins by interchanging regions between both paralogs in *Arabidopsis* and by creating chimeric genes consisting of a AthSPO11-backbone combined with parts from *Carica papaya*. In an additional experiment, we deleted and exchanged the last exon of both genes, since we discovered earlier that disruption of the respective protein in this part leads to a total loss of function. This indicates that it might be an essential part involved in the formation of a DSB-inducing complex, e.g., via binding of PRD1 [7,37,48].

## 2. Results

### 2.1. AthSPO11 is Located on the Chromosome during Leptotene and Early Zygotene

**Figure 3:**
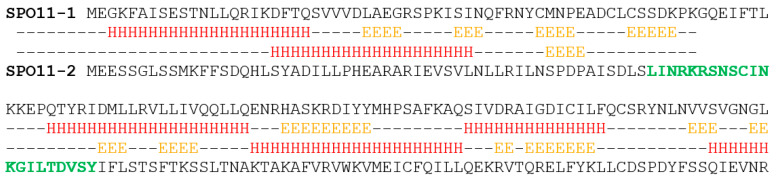
**JPred** [51] predicted secondary structure of A. thaliana SPO11-1 and SPO11-2. Predicted secondary protein structure of the two meiotic SPO11 paralogs in *A. thaliana* SPO11-1 (41.81 kilodalton) and SPO11-2 (43.13 kilodalton). Full length protein sequence is shown with subjacent predicted secondary structure. H = alpha helix; E = beta sheet; “–“ = random coil. The 21 amino acids that were used for the production of an N-terminal SPO11-2 antibody are shown in bold green.

To visualize the spatiotemporal localization of AthSPO11-2, we designed and produced a polyclonal antibody against AthSPO11 2 using a unique 21 aa long peptide localized in the N-terminal part of AthSPO11 2. The N-terminal part of the protein was selected since this part is highly variable between SPO11-1 and -2 in many plants and is predicted to be accessible by the antibody in localization studies (Figure 3).

**Figure 4:**
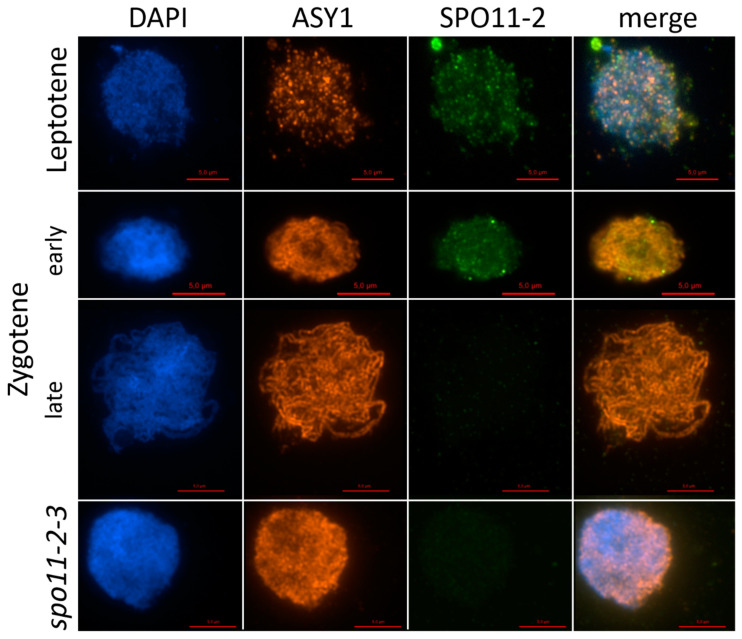
Dual immunolocalization of ASY1 and SPO11-2 proteins in meiocytes of *Arabidopsis thaliana* Col-0 and *spo11-2-3*. Meiocytes were counterstained with DAPI and dual immunolocalization of ASY and SPO11-2 was performed, single channel pictures were merged afterwards. Rabbit polyclonal IGg antibody against ASY1 and rabbit polyclonal IGg antibody against SPO11-2 was used. Meiocytes of Col-0 and *spo11-2-3* were used for immunolocalization. Bar = 5 µm.

The peptide was used to immunize mice and rabbits. The detection of SPO11-2 foci in immunolocalization spreads of pollen mother cells from wild-type *A. thaliana* anthers was possible with sera from all the immunized animals. The serum of the IM animals showed brighter signals; therefore, this serum was purged, and the purified antibodies were used in further studies. Immunolocalization studies using the purified antibody revealed the presence of foci during leptotene and early zygotene on the chromosomes (Figure 4). In the spo11-2-3 lines, no distinct signal could be detected, indicating that the antibody is specific against AthSPO11-2 and is not binding AthSPO11-1 or any other protein in a noticeable amount (Figure 4). Around 120 foci per cell could be identified on the chromosomes, which is comparable to the number of SPO11-1 foci found in wild-type A. thaliana plants ([46], personal communication). SPO11-2 can be detected during leptotene and the signals last until late zygotene, whereas SPO11-1 can only be detected during leptotene [46].

**Figure 5:**
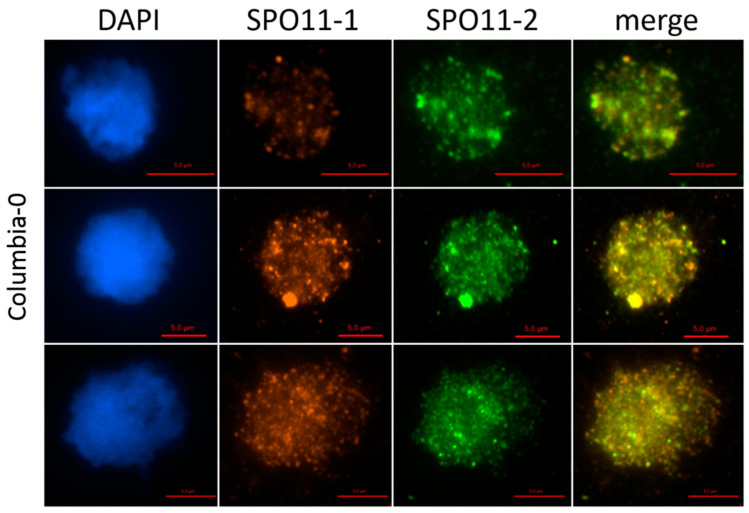
Dual immunolocalization of SPO11-1 and SPO11-2 proteins in meiocytes of Arabidopsis Col-0 plants. Meiocytes were counterstained with DAPI (A) and dual immunolocalization of SPO11-1 and SPO11-2 was performed, single channel pictures were merged afterwards. Mouse polyclonal IGg antibody against SPO11-1 and rabbit polyclonal IGg antibody against SPO11-2 was used. Bar = 5 µm.

Immunolocalization studies using antibodies against AthSPO11-1 and -2 revealed a colocalization of both signals in pollen mother cells during leptotene of prophase I (Figure 5).

Foci counting revealed a comparable number of foci/cells for SPO11-1 (124 ± 29; *n* =25) and SPO11-2 (128 ± 27; *n* =25) with a *p*-value of 0.31.

### 2.2. The Function of AthSPO11 Is Sequence Specific

To elucidate a sequence specificity of SPO11, we transformed *spo11-1-3* and *spo11-2-3* lines with *SPO11* constructs from different species. To exclude the fact that positional effects affect complementation and as a control, we transformed heterozygous *spo11-1-3*/SPO11-1-3 mutant plants with a full genomic construct of SPO11-1 from *A. thaliana*, including 553 bp of the promoter region and 496 bp of the 3’-UTR region (*spo11-1-3*-AthSPO1g). We did the same with heterozygous *spo11-2-3*/ SPO11-2-3 mutant plants, as we used the full genomic region of AthSPO11-2, including 704 bp of the promoter region and 496 bp of the 3´-UTR region (*spo11-2-3*-AthSPO2g). This architecture of the UTR regions was also used for all the other complementation approaches. We analyzed all the transgenic plants with a homozygous mutant background in the T0. Additionally, we analyzed complementation in the offspring of the heterozygotic mutant T0 plants. As in a previous study, most generated lines produced a similar number of seeds as the wild-type control and were able to fully complement the sterile phenotype of the respective knockout mutant (Table 1) [7].

**Figure 6:**
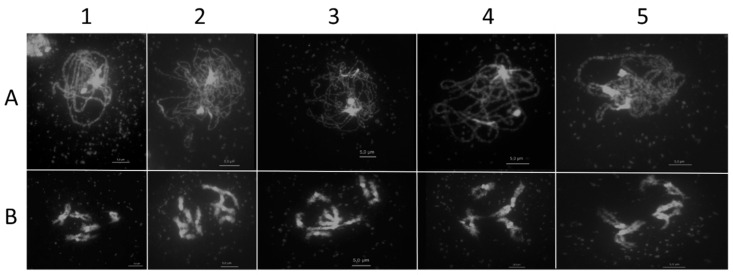
Light micrograph of DAPI-stained nuclei. Male meiotic chromosomes counterstained with DAPI during pachytene (A) and diplotene (B) stages of prophase I in wild-type (1) and homozygous *spo11-1-3* (2) and *spo11-2-3* (3) single mutants as well as homozygous single mutants complemented with endogenous SPO11-1 (4) and SPO11-2 (5) genomic DNA. Pairing of chromosomes during pachytene stage and formation of five bivalents could be observed in the case of wild-type and the complemented mutants. The single knockout lines do not show any pairing or formation of bivalents instead ten univalents are formed. Bar = 5 µm.

The analysis of the meiotic stages in the complemented plants showed a distribution and pairing of the chromosomes comparable to the wild-type control. The homologous chromosomes paired in the pachytene stage, and five bivalents were formed at the diplotene stage of prophase I (Figure 6).

**Figure 7:**
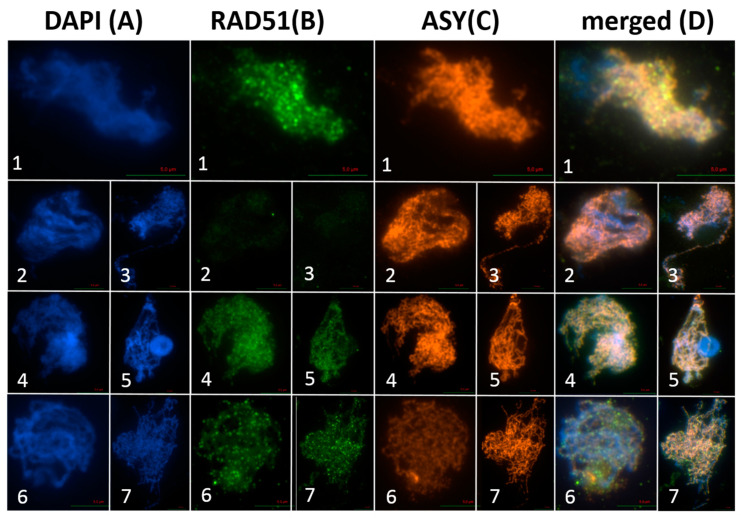
Dual immunolocalization of ASY1 and RAD51 proteins: Meiocytes were counterstained with DAPI (A) and dual immunolocalization of RAD51 (B) and ASY1 (C) was performed, single channel pictures were merged afterwards (D). Rabbit polyclonal IGg antibody against ASY1 and rat polyclonal IGg antibody against RAD51 was used. Meiocytes of Col-0 (1), *spo11-1-3* (2), *spo11-2-3* (3), *spo11-1-3*-AthSPO1g (4), *spo11-2-3*-AthSPO2g (5), Col-0-AthSPO1g (6) and Col-0-AthSPO2g (7) were used for immunolocalization. Bar = 5 µm.

Immunolocalization studies in the spread preparations of *spo11-1-3*-AthSPO1g and *spo11-2-3*-AthSPO2g meiocytes revealed a restoration of RAD51 loading onto the DNA (~150 foci/cell; *n* = 10) (Figure 7). In *spo11-1-3* and *spo11-2-3*, RAD51 foci could not be detected (Figure 7). The additional expression of a respective SPO11 under its natural promoter in Col-0 wild-type plants had no influence on the number of DSBs, since the number of RAD51 foci remained comparable to the wild-type control (~150 foci/cell; *n* =10) (Figure 7).

To investigate the sequence specificity of SPO11, we created constructs with interchanged parts between AthSPO11-1 and AthSPO11-2 (named SPO1swap1 to 3 and SPO2swap1 to 3) but kept the respective endogenous promoter and 3´-UTR. In the swapped regions, both proteins showed less sequence identity between each other compared to the conserved parts of the proteins (Supplementary Figure S1). We especially exchanged the N-terminal part between SPO11-1 and SPO11-2, as this part seems to be involved in the complex formation with MTOPVIB [42]. We analyzed multiple lines of each construct transformed in the *spo11-1-3* and *spo11-2-3* background. None of the lines showed any successful complementation (Table 1). All the lines showed the same reduced seed set as the respective SPO11 knockout control.

The C-terminal end seems to have an essential function for AthSPO11-1 and AthSPO11-2, since the disruption of this part of the protein, namely the last exon, by T-DNA insertion is leading to a complete loss of function for AthSPO11-2. Additionally, the last exon is quite conserved between both proteins since it is containing the seven conserved motifs and ten additional conserved amino acids (Figure 1). We wanted to elicit if the loss of function due to the T-DNA insertion is caused by disrupting the overall structure of the protein or if the loss of the last exon alone has the same devastating effect on AthSPO11-2 as well as on AthSPO11-1. For this purpose, we designed full genomic constructs for AthSPO11-1 and AthSPO11-2 lacking the last exon. An artificial stop codon (TAG) was introduced just after the penultimate exon of AthSPO11-1 and AthSPO11-2. The gene specific 3´-UTR region was fused to this artificially truncated protein (SPO1Δlex and SPO2Δlex); none of the transformed lines showed an increased number of seeds. (Table 1) To address the question whether the function of the last exon is conserved between AthSPO11-1 and AthSPO11-2, we additionally interchanged the last exon between both genes (SPO1swap4 and SPO2swap4) (Figure 1). Four lines of SPO1swap4 showed a slightly but significant induction of seed production per plant compared to the control knockout line (8.5% ± 0.8 vs. 3.5% ± 0.5 *p*-value > 0.001 *n* = 10). This effect was not observed for SPO2swap4 Table 1). After the transformation of the constructs in the opposite genetic background, no obvious change in fertility could be observed.

The expression of the interchanged constructs had, in most cases, no influence on the seed set at all, neither in the homozygous mutants nor in the corresponding heterozygous or wild-type plants. Only for SPO1swap2 was a reduced seed set in wild-type plants observed in many lines.

### 2.3. The Function of SPO11 Is, to a Certain Extent, Species Specific

To investigate if the function of SPO11 is conserved between differently related plants, we tried to complement the sterile phenotypes of *spo11-1-3* and *spo11-2-3* with genes from various species. In a heterologous complementation approach, the full genomic sequence of SPO11-1 and -2 from rapeseed (*Brassica rapa*), papaya (*Carica papaya*) and rice (*Oryza sativa*) were used. All the constructs were fused with the promotor and 3’-UTR of the corresponding *A. thaliana* gene as mentioned above. *B. rapa*, which is closely related to *A. thaliana* (~20 mya), shows the highest sequence identity, followed by *C. papaya*, which diverged from *A. thaliana* around 72 mya [52,53] (Table 2). Rice diverged earlier, during the monocot/dicot split, which ranges back to 150 to 200 mya and shows less sequence identity [54]. After the split, the sequence of SPO11 changed between both groups; 19 additional amino acids are coded in SPO11-1 in monocotyledonous plants, which cannot be found in any dicotyledonous plant analyzed thus far (Table 2).

**Figure 8:**
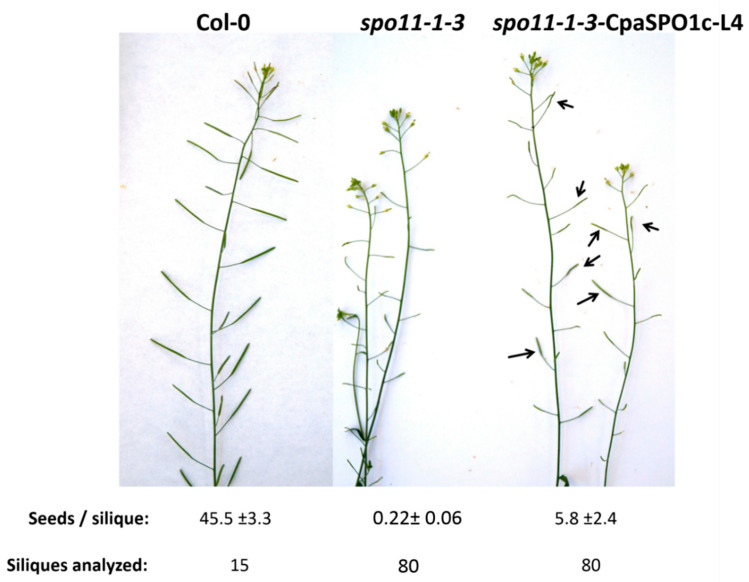
Comparison of wild-type Col-0 with homozygous *spo11-1-3* and spo11-1-3-CpaSPO1c Line 4 inflorescence. Enlarged siliques with intact seeds in the complemented line are marked with an arrow.

Several individual *spo11* lines carrying a genomic SPO11 construct from *B. rapa, C. papaya* or rice were analyzed. Nearly all of the lines carrying SPO11 from *B. rapa* showed full complementation of the sterile phenotype of the corresponding mutant (Table 3). However, none of the lines carrying a construct of papaya or rice SPO11 showed any complementation at all. All the lines had the same reduced seed set as their respective knockout control (Table 3). When analyzing the expression of the inserted genes in *Arabidopsis* flowers in detail, no correct spliced isoform of CpaSPO11-1 could be identified. To investigate if the splicing of SPO11 was the reason for the failed complementation efficiency in the complementation approaches, the cDNA of SPO11-1 and SPO11-2 from *Arabidopsis*, rapeseed and papaya has been used for complementation approaches in the same way as the genomic constructs. Similar to the constructs using the genomic DNA, most generated lines carrying SPO11 from *Arabidopsis* and rapeseed in a *spo11* background produced a similar number of seeds as the wild-type control. However, in contrast to the genomic complementation approach, two lines carrying multiple copies of a papaya SPO11-1 cDNA complementation construct also showed an increase in seed production (Figure 8).

**Figure 9:**
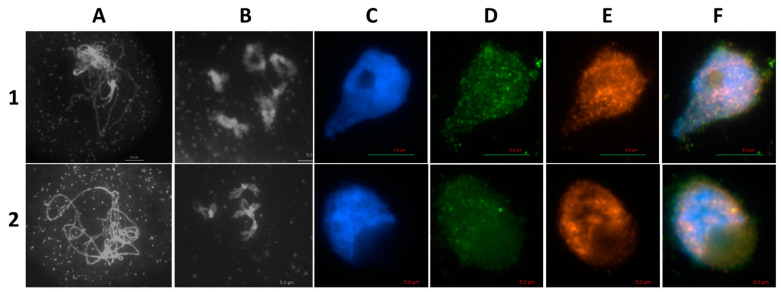
DAPI images and dual immunolocalization study of ASY1 and RAD51: Male meiotic chromosomes counterstained with DAPI during pachytene (**A**) and diplotene (**B**) stages of prophase I in wild-type (**1**) and homozygous spo11-1-3-CpaSPO1c Line 4 (**2**). Meiocytes were counterstained with DAPI (**C**) and dual immunolocalization of RAD51 (**D**) and ASY1 (E) was performed, single channel pictures were merged afterwards (**F**). Rabbit polyclonal IGg antibody against ASY1 and rat polyclonal IGg antibody against RAD51 was used. Bar = 5 µm.

We could detect a total seed set of around 40% compared to the wild type for line four, which carries multiple copies of the transgene. The average seed number/silique was still reduced with ~5.8 ± 2.4 seeds per silique (*n* = 80) but the total number of siliques per plant was increased, similar to the respective SPO11-1 knockout line, *spo11-1-3*. The DAPI stained spreads of the pollen mother cells of this line showed pairing of the chromosomes at the pachytene stage in ~20% of the cells. The formation of bivalents during the diplotene stage could also be observed in some cases (Figure 9). Immunolocalization studies in the spread meiocytes of *spo11-1-3*-CpaSPO1c line 4 showed that in some cells, the loading of RAD51 onto the chromosomes was restored (Figure 9). The number of foci per cell is highly variable, making it impossible to give a meaningful mean. By analyzing the offspring of this line, we discovered a restoration of siliques and a 10 times higher seed number per silique compared to the respective knockout line (Table 3). The complementation with SPO11-2 cDNA from papaya as well as the attempt to complement with a combination of papaya SPO11-1 and -2 cDNA failed. When analyzing the expression of papaya *SPO11* genes in Arabidopsis, a high rate of incorrect spliced cDNA missing multiple exons (e.g., exon 3 and 4) have been detected for CpaSPO11-1 and -2. To elucidate whether the interchanged SPO11 constructs between papaya and *Arabidopsis* are functional, we exchanged the N- and C-terminal parts of *Arabidopsis* with the ones from papaya. The exchanged parts include the first three exons for the N-terminal exchange and the last exon for the C-terminal exchange of SPO11-1 and -2, respectively. A complementation approach has not been successful, neither for genomic DNA nor for cDNA, as all the clones showed the same reduced seed set as the knockout control.

### 2.4. The Splicing Landscape of SPO11 Homologs Changes when Transformed in A. thaliana

**Figure 10:**
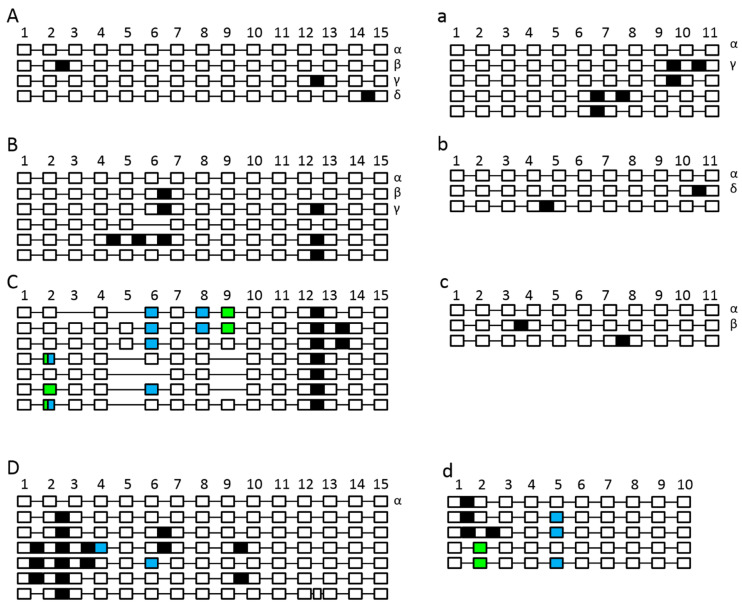
Schematic unscaled schema of different splice forms of SPO11-1 (**A**–**D**) and -2 (**a**–**d**). *Arabidopsis thaliana* (**A**,**a**), *Brassica rapa* (**B**,**b**), *Carica papaya* (**C**,**c**) *and Oryza sativa* (**D**,**d**). SPO11-1 (**A**,**B**,**C**,**D**) and -2 (**a**,**b**,**c**,**d**) have been reintegrated into *Arabidopsis*. Exons are numbered and shown as white blocks, spliced introns as black lines. Intron retention events are illustrated as black boxes, alternative 5′splice site selection are shown as blue boxes and alterative 3′splice site selection as light green boxes. In the case of exon skipping, the corresponding white box is missing. Previously known splicing forms are named in Greek letters, see [33].

In previous studies, it has been identified that SPO11 is differentially expressed and spliced in plants [32,33]. In this study, it was elicited whether the aberrant splicing is sequence and/or species specific and if the splicing is affected by neighboring sequences. By examining the different splice variants of both AthSPO11 transformed in the corresponding mutant background, no unknown splice variants for AthSPO11-1 could be detected. Nevertheless, three differentially spliced transcripts, all retaining introns (splice variants β, γ and λ) besides the functionally spliced form (Figure 10A), were present. When analyzing the splicing behavior of AthSPO11-2 in its corresponding mutant, four additional transcripts, which all showed intron retention (IR) besides the functional spliced variant, have been identified. One of these forms was known (γ) and three were previously unknown (Figure 10a).

**Figure 11:**
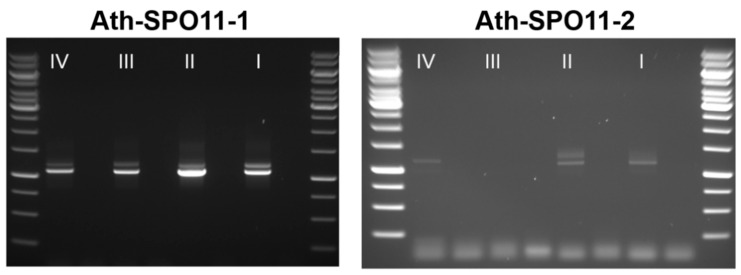
Semiquantitative RT-PCR of SPO11-1 and -2 from *Arabidopsis thaliana*. Different Anthere stages (I–IV) have been evaluated for alternative splicing using a semiquantitative RT-PCR using the same amount of RNA. In most samples, the alternative or aberrant splicing products are visible as an additional band in the gel.

By analyzing the Col-0 wild-type control, four additional, previously unidentified spliced transcript variants of AthSPO11-2 have been identified. We analyzed the temporal distribution of the alternative splicing patterns of SPO11-1 and SPO11-2 in the following four different stages of anthers (Figure 11): (i) premeiotic (Stages 1–3 according to Sanders et al. [55]), (ii) meiotic (Stages 4–6), (iii) post meiotic (Stages 7–9) and (iv) ripe pollen (Stages 10–12). By performing a transcriptome sequencing analysis, we have been able to confirm at least most of the intron retention events for both SPO11-1 and -2 (Supplementary Figure S2). We could detect the highest expression of SPO11 in the meiotic stages and the highest amount of differential spliced transcript variants in the pre-meiotic and meiotic stage (Figure 11).

The expression in the post meiotic and ripe pollen were considerably lower, as in the pre-meiotic and meiotic ones. By analyzing 50 sequences from each stage, we could detect a differential splicing rate of 45% for *SPO11-1* and 33% for *SPO11-2* in stage one. In stage two, the rate was 25% for *SPO11-1* and stayed as 33% for *SPO11-2*. For stages three and four, not enough sequences could be cloned for *SPO11-2*, but for *SPO11-1*, the differential splicing rate dropped down to 8%. In the premeiotic and meiotic stages, many of the differential spliced transcripts showed alternative 5 and 3’ splicing resulting in functional but minimal shortened versions of *SPO11-1* and *-2* (-1 or -3 AA). Furthermore, intron retention could be observed, mainly intron 12 for *SPO11-1* and *-2* mainly Intron 6 and 7. In the later stages, the retention of Intron 2 could be observed for SPO11-1 as well.

When analyzing the aberrant splicing of BraSPO11-1 in *A. thaliana*, we could detect five aberrant spliced transcript variants besides the correct spliced form. We identified two previously known splice variants from *B. rapa* (β and γ) as well as two previously unknown forms, one IR and one splice form in which exon six was skipped. The fifth detected form is a retention of intron 12 and identical with the splice variant γ from *A. thaliana*. For BraSPO11-2, we could detect, besides the functional form, two additional transcript variants; one was previously identified in *B. rapa* (δ) and one new variant, in which intron four was retained (Figure 10B,b).

The splicing landscape of CpaSPO11-1 in *A. thaliana* is divergent, as it is in *C. papaya* itself. We detected seven previously unknown spliced transcript variants, all containing intron 12 in a combination with alternative 5’- and/or 3’-splice site selection, exon skipping and the retention of additional introns (Figure 10C). A functional spliced form of CpaSPO11-1 could not be detected in the flowers of *A. thaliana*. The splicing of CpaSPO11-2 in *A. thaliana* resulted in two additional aberrant spliced transcripts beside the functional form, one of the transcript forms is known from *C. papaya* (β) and one is comparable to one found for AthSPO11-2 (κ) (Figure 10c).

The splicing landscape of OsaSPO11-1 in *A. thaliana* is also disturbed, as we were able to identify the correct spliced variant only in a very low amount, but we detected at least six aberrant spliced transcript variants, all showing IR. Three variants additionally showed an alternative 3-splice site selection and in one variant a completely new exon between exon 12 and exon 13 could be identified (Figure 10D). The splicing of OsaSPO11-2 showed miss-splicing in all the analyzed variants. We identified a complete or partial retention of intron 1 or 2 or both in all the analyzed cases, leading to early termination due to premature stop codons (Figure 10D).

**Figure 12:**
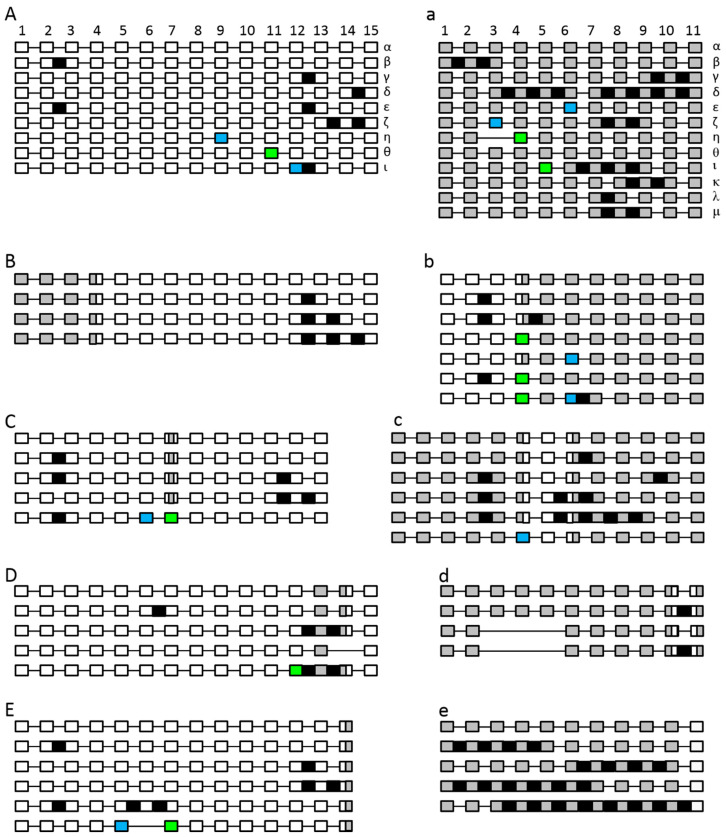
Schematic unscaled schema of the different splice forms of SPO11-1 (**A**–**E**) and -2 (**a**–**e**). *Arabidopsis thaliana* WT (**A**,**a**), *Arabidopsis thaliana* Swap1 (**B**,**b**), *Arabidopsis thaliana* Swap2 (**C**,**c**), *Arabidopsis thaliana* Swap3 (**D**,**d**) and *Arabidopsis thaliana* Swap4 (**E**,**e**) reintegrated into *Arabidopsis*. Exons are numbered and shown as white (SPO11-1) or grey (SPO11-2) blocks, spliced introns as black lines. Intron retention events are illustrated as black boxes, alternative 5’splice site selections are shown as blue boxes and alterative 3’splice site selections as light green boxes. In the case of exon skipping, the corresponding white box is missing. Previously known splicing forms are named in Greek letters, see [33].

Additionally, we investigated the aberrant splicing of the swapped constructs. In most cases, a correct splicing of the corresponding construct was observed (Figure 12). Aberrant splicing was also observed but most events are related to the corresponding SPO11. Most of the aberrant splicing of AthSPO11-1 parts was previously described for AthSPO11-1 and most of the aberrant splicing of AthSPO11-2 parts was also described before for AthSPO11-2 (11). Nevertheless, some new splicing patterns have been found for both genes, especially when the C-terminal part was changed.

## 3. Discussion

In most known eukaryotic organisms, a proper pairing of homologous chromosomes with subsequent recombination via crossovers is essential for genetic variability as well as proper disjunction of the chromosomes in the first meiotic division. Eukaryotic SPO11 plays a major role in the induction of meiotic DSBs and without those no pairing of chromosomes and subsequent random disjunction occurs. The ancestral “SPO11”, TOPVIA from *archaea* is working in a tetrameric complex, composed of two TOPVIA and two TOPVIB subunits each. The protein complex is able to cut and relegate DNA double strands in one process. Fungi and mammals contain and use only a single homolog of TOPVIA and seem to have lost the second subunit. The SPO11 proteins have kept their ability to cleave double stranded DNA but the resealing of the breaks has been taken over by other proteins such as DNA ligase IV in combination with XRCC4 or XRCC3 [56,57,58,59]. In contrast to mammals and fungi, land plants have kept two TOPVIB homologs as well as at least three TOPVIA homologs (in *Arabidopsis* SPO11-1, -2- and -3) [13,33,42]. In *Arabidopsis*, an interaction with TOPVIB has been shown for SPO11-3 in mitosis and for MTOPVIB-like with SPO11-1 and -2 in in vitro studies [32,42]. Even though it is a subject of general interest, no solution is present for the question, why do plants need and encode for two meiotic active SPO11? Due to a number of studies performed on AthSPO11-1 and AthSPO11-2, it is known that the function of both proteins is not redundant [7,12,37]. At least one functional copy of each SPO11 is needed for proper meiosis in *A. thaliana* and wheat [49,50]. Furthermore, it remains unclear whether and how these two interact in vivo and which regions of the protein are essential. Alterations of single AA in the non-conserved parts of SPO11 seem to have no negative effect on the DNA binding activity and sometimes do not even alter the cleavage capability of SPO11-1 [60]. With our heterologous complementation approach, we wanted to evaluate if the function of SPO11 is conserved between orthologous SPO11 genes from organisms that are related to a different extent. Since a fully functional complementation is possible between both AthSPO11 and BraSPO11, the function seems to be conserved at least in the family of the *Brassicaceae*. Multiple small changes, especially in the N-terminal part of SPO11-1 and -2, seem to have no negative effect on its function, since this is the part where SPO11 from *A. thaliana* and *B. rapa* differ most. The overall structure seems not to be influenced by these small changes, as SPO11 from both species must have the conserved domains at the appropriate location for a functional interaction. The subsequent repair of the breaks is conducted as in wild type, indicating that the putative interacting factors of AthSPO11 can also recognize BraSPO11. If not, the fragmentation of the chromosomes should be visible just as it is known from mutants lacking DSBs’ repair proteins such as MRE11 or RAD51 [61,62]. A positive complementation approach with CpaSPO11 is not possible under natural expression conditions using genomic DNA, even though the sequence identity is quite high (~73%). This is in contrast to successful complementation approaches using wheat SPO11-1 and -2 [49,50]. However, in these experiments, cDNA under the control of a strong promoter has been used and, therefore, we speculate that aberrant splicing prevents complementation. After evaluating the splicing landscape of CpaSPO11-1 in *Arabidopsis,* we detected a divergent pattern of aberrantly spliced forms, as it is the case in papaya [33]. A functional spliced form of CpaSPO1g could not be detected in flowers of *Arabidopsis*, but the presence of a functional spliced form is not excluded, since in papaya this form is very rare, too. The multiple insertions of CpaSPO1 cDNA were leading to an increased number of seeds. This is somehow comparable to the complementation achieved by wheat SPO11-1 and -2, but not absolutely. In our analyses, we used the natural promoter and 3´-UTR of both genes in the wheat studies, a ubiquitin promoter for TaeSPO11-1 and the RAD51 promoter for TaeSPO11-2 was used. As the natural promoters are weak (especially for AthSPO11-2) and meiosis specific, the mRNA, in case of the heterologous complementation, is a) already properly spliced (in case of cDNA) and b) strongly expressed (in case of the ubiquitin promoter). The data from Benyahya et al. and Da Ines et al. showed a very good complementation, but it was somehow partial except for the wheat cDNA in rice that showed full restoration [49]. This can explain why we did not achieve complementation using the most natural situation and the full genes but a partial complementation when several copies of the cDNA from papaya were found in *Arabidopsis*. It is reasonable to speculate that somewhere in the gene sequence (in the introns or even in the promoter), signals for aberrant and alternative splicing exist, that lead to only a small amount of full length and functional mRNA. In a case of heterology of the sequences, it might lead to the disastrous splicing events as we detected for papaya SPO11 genes in *Arabidopsis*. Furthermore, the only partial complementation with cDNA from papaya might have additional reasons; (i) the binding of CpaSPO11-1 on the DNA of *A. thaliana* is not effective enough due to changes in the TOPRIM and or winged helix domain. When it is expressed multiple times, the loading of CpaSPO11-1 onto the DNA might be enough to create a sufficient number of breaks, ensuring the pairing of DNA in some cells. In other cells, there might be an insufficient number of breaks as no pairing is visible. (ii) Cpa SPO11 is binding to the DNA but a break cannot be induced by the insufficient binding of partner proteins or improper binding to the respective second SPO11 protein to build up a complex. We cannot dismiss the last possibility because a combined expression of CpaSPO11-1 and -2 was not leading to an enhanced seed set, but there might be other proteins necessary that coevolved with SPO11 in each plant and, therefore, cannot recognize the ones from papaya.

One possibility to obtain a better understanding of what happens is to produce an antibody against CpaSPO11-1 and have a look on its distribution to see if a loading of CpaSPO11-1 onto the DNA, as it is known from AthSPO11-1, could be seen [46]. A positive complementation approach could not be observed for CpaSPO11-2, the reason is unclear and hard to explain since the sequence identity between SPO11-2 from papaya and *Arabidopsis* is higher than between the orthologous SPO11-1 proteins. One possibility is that we did not have enough CpaSPO11-2 loading onto the DNA to create a break since we have not had a line with multiple copies integrated and the AthSPO11-2 promotor is very weak. Creating such a line and having a look on its meiocytes could answer this question.

Further, we addressed the question of whether the function of each SPO11 is sequence specific and encoded in the respective non-conserved parts. By exchanging these parts of the respective SPO11, we hoped to identify regions of SPO11 that are defining the differences between both SPO11 in *A. thaliana*. Since we could not observe any difference in the seed set between the complementation approaches using swapped constructs and the respective knockout lines, we have to assume that the species specificity of SPO11 lies in more than one region. Nevertheless, we cannot rule out that the exchange of sequence parts is leading to a disruption of the overall structure of the whole protein or to a disruption of a specific domain. In both cases, a functional interaction might be prevented. We have not modified the structure of the very conserved TOPRIM domain by exchanging parts of it, which is assumed to span from motif three to motif five within the swapped approaches [63]. However, the CAP domain, including the winged helix domain, is disrupted, at least by the first swap, and it seems that such an exchange is not functional. Additionally, the winged helix domain, which is located ranging from aa 9 to 137 in Ath SPO11-1 and between aa 91–163 in AthSPO11-2, is disturbed, too. This region has been found to be the interaction partner of MTOPVIB-like in Y2H assays.

The second swap approach, which is the smallest one, is not harboring any conserved motifs. It also showed no positive complementation but a reasonable negative interference on wild-type and heterozygous plants could be observed. Such an effect of partial sterility, but not in such a severe manner, was observed earlier for SPO11 genes mutated in their active tyrosine residue [7] but never for wild-type genes, showing that it is functional and does not control for position effect. These finding allow us to suggest that SPO1swap2, controlled by its native promotor as well as 5’ and 3’ region, seems to bind to the DNA but is not able to cleave it, which might have different possible reasons such as (i) the misfolding of the chimeric SPO11 protein and physical distortion of the DNA/SPO11-1 and -2 cleavage complex, (ii) the disability of binding interaction partners that are necessary for cleavage and (iii) the tighter binding of interaction partners paired with a missing ability to cleave DNA, which results in their sequestration into an inactive complex. In all of these possible cases, the chimeric protein stands in competition to the natural occurring SPO11. Further analyses have to be performed to investigate the possible structure of this chimeric protein, for example, by mutating single aa and investigating the binding capacity of DNA as it was performed before for SPO11-1 [60].

With this publication, the first direct detection of SPO11-2 foci on the chromosomes could be shown. The fact that we could not observe distinct foci in spo11-2-3 lines shows that the antibody is specific enough to detect SPO11-2 only. We could observe a comparable number of SPO11-1 and -2 foci on the DNA, which were mostly overlapping, during the early leptotene stage of meiosis, giving a hint that both proteins seem to be located at the same spot during leptotene. Nevertheless, we could observe that SPO11-2 foci were detectable even during the zygotene stage, whereas SPO11-1 foci disappeared already in late leptotene. However, in other organisms, such as mice and yeast, such a behavior of SPO11 staining was observed before [63,64]. The function of that late detectable SPO11-2 on the chromosome is still ambiguous, since DSB induction is clearly induced earlier. Therefore, it can only be speculated that the presence of SPO11 in later stages might have other function than cleaving the DNA. Prieler et al. [64] suggested an interaction of these late SPO11 with recombination hotspots. However, in plants such an interaction was never shown before and further analyses, such as the detection of SPO11-2 on SPOligos in combination with hotspot identification, are necessary.

The analysis of the splicing landscape of the different orthologous plant SPO11 genes in *Arabidopsis* showed that there is a species-specific pattern of aberrant splicing for SPO11. Since SPO11 from closely related plants was spliced predominantly in a correct way, the splicing of SPO11 from more distant plants seems to be much less effective. New splicing patterns were found, especially for the SPO11-1 of papaya and rice, which had never been observed thus far. This allowed us to suggest that the splicing of SPO11 is not only embedded in the plain sequence of the respective SPO11 gene but also in other factors. One possibility could be that the native SPO11 promotor from *Arabidopsis* has some regulatory sequences that could influence the splicing patterns or that other factors exist that remain unknown and are probably species specific. Taking a closer look on SPO11 splicing in various plants as well as at different time points during meiosis would be of great interest to gain a better understanding of this putative regulation step of SPO11 by aberrant and maybe alternative splicing patterns. In mice and humans, two distinct splice variants of SPO11 have been identified that possess different features concerning the timing of the DSB induction, as studies in mice have shown [27,28]. It is proposed that SPO11 forms multimers or dimers between itself and/or between distinct spliced variants, this could also be possible in plants as we and others have now identified potential functional spliced variants of SPO11-1 and -2. In particular, the N-terminal region seems to be of great importance, as many of the differential splicing and intron retention happens there [33,65] this study.

## 4. Materials and Methods

### 4.1. Plant Material and Growth Conditions

For the complementation approaches, the mutant lines spo11-1-3 (SALK_146172) and spo11-2-3 (GABI line 749C12) were used. Both mutant lines have been previously described [7,37,66]. For propagation and to obtain anthers for evaluation of meiosis in pollen mother cells, the plants were grown as previously described [33]. For the selection of positive transformation events, seeds from inflorescence transformed with *Agrobacterium tumefaciens* were surface sterilized with 4% sodium hypochlorite, stratificated at 4 °C overnight and sown on agar plates containing germination medium (GM = 4.9 g/L Murashige and Skoog including vitamins, 10 g/ L sucrose and 0.8 g/ L agar (adjusted to pH 5.7 with KOH)). The plantlets were cultivated in a plant culture chamber under controlled conditions of 22 °C with 16 h light and 8 h dark.

### 4.2. Molecular Characterization of the Mutant Lines

For genotyping of the mutant plants, DNA was extracted from a small leaf of the plantlets. For PCR analysis, first primer pair was used to amplify the sequence that is interrupted by T-DNA in the mutants (SPO11-1: SP1-2 and SP1-R3; SPO11-2: SP2-2 and SP2-RP2) The presence of the T-DNA insertion was checked using a left border specific primer for each line (SALK LBd1 for SPO11-1 and GABI LB1 for SPO11-2) and a gene specific primer for each SPO11 gene located downstream of the T-DNA insertion (SP1-R3 and SP2-RP2). Transformed plants were double checked by growing on media containing 6 mg/l phosphinothricin (PPT) and PCR checked using a primer pair specific for the insertion of the phosphinothricin acetyl transferase (PAT). To identify the genetic background of the plants transformed with SPO11 from *A. thaliana*, *Brassica rapa* and papaya (*Carica papaya*), a primer pair located outside the promoter and 3’-UTR region was used (SPO11-1: SP1-3Lr2 and SP15L4; SPO11-2: SP2-(-5) and SP2-R (-4)) to amplify the sequence that is interrupted by the T-DNA, due to high sequence identity between the endogenous SPO11 and the introduced paralog SPO11 gene.

### 4.3. Protein Prediction

The prediction of the secondary structure in Figure 3 was made using Jpred V. 3.0 [51].

### 4.4. Plasmid Construction and Plant Transformation

Transformation of *A. thaliana* was performed as described [67]. Due to the sterility of homozygous *spo11* mutants, plants heterozygous for the T-DNA insertion had been used for transformation. The constructs used for plant transformation are based on the binary plasmid pPZP201 [68] with an enhanced multiple cloning site (MCS) and modified as previously described [69]. For the double mutants, *spo11-1-3*:SP1Pro:CpaSPO11-1 SP2Pro:CpaSPO11-2cDNA and *spo11-2-3*: SP2Pro:CpaSPO11-2 SP1Pro:Cpa SPO11-1cDNA, the vector was edited, and the PPT-resistance cassette under the control of the CaMV 35S gene promoter was exchanged by a gentamycin resistance gene (aaaC1) under the control of the PcUbi4-2 promoter. Plants homozygous for the first event were used for the transformation with the second gene. After the selection of transformed plants in the T1 generation by PPT resistance (6 mg/L PPT), the T2 generation was checked for mendelian 3:1 segregation to obtain lines with a single insertion event.

### 4.5. RNA Extraction and RT-PCR

All kits used in this study were applied, if not especially mentioned, strictly following the manufacturer’s instructions. Total RNA of *A. thaliana*, *B. rapa* and papaya was isolated from fresh young flowers using the RNA mini Kit from Bio and Sell (Bio and Sell e.K., Feucht, Germany). Isolated RNA was treated with DNase I (Thermo Fisher Scientific, Dreieich, Germany) and afterwards cleaned and concentrated using the GeneJET RNA Cleanup and Concentration Micro Kit (Thermo Fisher Scientific, Dreieich, Germany). To check contamination with genomic DNA in the DNase I treated RNA, a PCR was performed with RNA as template using gene specific primers for SPO11-1. cDNA was produced using an anchored oligo-dT Primer (VT20) using the Maxima H Minus Reverse Transcriptase Kit (Thermo Fisher Scientific, Dreieich, Germany) using 5 µg of total RNA as a template for RT-reaction.

### 4.6. Molecular Characterization of SPO11 Splice Variants

The screening for aberrant spliced SPO11 transcripts were performed as previously described, with one exception [33]. For analysis of the splice variants of the transgene, plants homozygous for the respective knock out were used for RNA isolation and cDNA production, to ensure that contamination with the endogenous transcripts was excluded.

### 4.7. Complementation Experiments

We generated several constructs to rescue the observed phenotypes of spo11-1-3 and spo11-2-3 and to check whether a heterologous SPO11 protein is able to complement the sterility. For all complementation approaches, the respective promoter and 3’-UTR region of the corresponding SPO11 were used as described [7]. The genomic regions from ATG to Stop of SPO11-1 and -2 from *A. thaliana, B. rapa*, papaya and rice (*Oryza sativa*) were amplified using gene specific primers with a 15 bp long attached linker by a high proofreading polymerase (Q5^®^ High-Fidelity DNA Polymerase, New England Biolabs, Ipswich, MA, USA). Linker sequences were homolog to the corresponding 5´- and 3´-UTR-region from the respective SPO11 from *A. thaliana*. The corresponding promoter and 3´-UTR regions were added to the heterologous genes and transferred into the binary vector via the homologous linkers using the In Fusion High-fidelity Cloning Kit (Takara Bio Europe/Clontech, Saint-Germain-en-Laye, Yvelines, France). The cDNA constructs for SPO11-1 and SPO11-2 from *A. thaliana*, *B. rapa* and papaya were amplified using high quality cDNA samples prepared from fresh young flowers, also using the same linker primers as for the genomic DNA. All amplified genes were fully sequenced after construction and before we transformed them into the corresponding heterozygous mutants.

Constructs with interchanged section between AthSPO11-1 and -2 were prepared using segment specific primers with attached linkers. Resulted fragments were fused together by the sites of homology and added to the vector using also the In Fusion High-fidelity Cloning Kit. Seed set was calculated as mean ± SEM of each genotype by comparing the mean number of seeds from wild-type and heterozygous plants of the individual construct with every plant carrying the respective construct.

### 4.8. Preparation of Pollen Mother Cells

The staining of the chromosomes of the pollen mother cells was performed as described [70]. Primary inflorescences were cut just after the first bud had opened and were fixed in ice cold fixative (3:1 ethanol: acetic acid). After 24 h, the fixative was exchanged. Flowers were dissected in fixative under a stereo microscope. All buds containing mature pollen were discarded, all other buds were washed 3× in 0.01 M citrate buffer (pH 4.5) and digested in a mixture of 0.33% cellulase (C1794, Sigma-Aldrich Chemie GmbH, Taufenkirchen, Germany) and 0.33% pectolyase (P5936, Sigma-Aldrich Chemie GmbH, Taufenkirchen, Germany) in 0.01 M citrate buffer for 90 min at 37 °C in a humid chamber. Each flower bud was squashed on a separate slide, mixed with 5µL of 60% acetic acid, briefly stirred and incubated for 45 s on a heated plate at 45 °C. A ring of fixative was drawn around the droplet and the slide was tilted, afterwards the slide was dried from the back using a hairdryer.

Meiocytes for immunolocalization of meiotic proteins were isolated, with minor changes as described [70] from immature flower buds, using stereomicroscope and fine forceps. Inflorescence was cut and immediately put on a damp filter paper. All buds containing mature pollen were discarded from the other buds, anthers were dissected transferred onto a clean glass slide (~40/slide) and digested in 5 µL of a mixture of 0.4% cytohelicase (C8274, Sigma-Aldrich Chemie GmbH, Taufenkirchen, Germany), 1.5% sucrose and 1% of polyvinylpyrolidone K30 (MW 40,000; Carl Roth GmbH and Co. KG, Karlsruhe, Germany) for 5 min on a heated plate at 37 °C in a humid chamber, smashed and digested in additional 5 µL of digestion medium for 5 min. Afterwards, this was mixed with 10 µL of 0.25% of Lipsol (SciLabware Limited, Staffordshire, UK) as spreading medium, incubated for 4 min at 37 °C on a heated plate and then fixed for min. 2 h with 4% paraformaldehyde under the fume hood. Immunostaining was performed as described using antibodies (ABs) against the meiosis specific protein AthASY1 (1:1000) and AthRAD51 (1:200) [46,71,72]. As secondary ABs, 1:200 goat anti-rat conjugated with Alexa488^®^ (112-545-167, Dianova GmbH, Hamburg, Germany) and 1:200 goat anti-rabbit conjugated with Cy3 (111-165-144, Dianova GmbH, Hamburg, Germany) ABs were used.

All slides were stained with 7 µL of VECTASHIELD antifading mounting medium (H-1000, Vector Laboratories Inc., Burlingame, CA, USA) containing 0.01 mg/mL DAPI (4´6-Diamidin-2-phenylindol). Staining of chromatin and meiotic stages was analyzed using a fluorescence microscope (Nikon ECLIPSE Ni-E microscope, DAPI-5060C; CFI Plan Apochromat 60X/1.4 and 100X/1.45, DS-QiMC camera, Minato, Japan).

### 4.9. Statistical Analyses

Statistical analyses were performed using Welch´s T-Test.

### 4.10. Protein Alignments

Proteins were aligned using Clustal Ω analysis in Lasergene 14 using standard configs.

### 4.11. Antibody Production

The production of the antibodies was performed by the group of Dr. Frank Rabenstein from the Institute for Epidemiology and Pathogen Diagnostics of the Julius Kuehn Institute, using rabbits from an undefined strain and mice from the BALB/c strain. Peptide was applied intravenously (IV) and intramuscularly (IM) in rabbits and subcutaneously in mice. Immunization of rabbits by IV injection of the corresponding peptide (GenScript USA Inc., NJ, USA) was induced by five injections of the peptide in a two-day interval, injecting 60 μg of peptide in 0.9% sodium chloride solution two times, followed by 90 μg two times and 120 μg once. Three blood samples were taken on a weekly base, starting three weeks after the first injection. IM immunization of rabbits was induced by injection of 400 μg of peptide mixed one to one with Freund´s complete adjuvant, followed by two injections of 400 μg of peptide with Freund´s incomplete adjuvant after three weeks each. Three blood samples were taken; the first was taken ten days after the last injection, followed by two blood samples taken on a weekly base. Mice were immunized by subcutaneous injection of 100 μg of peptide mixed one to one with Freund´s complete adjuvant, followed by one injection of 100 μg of peptide mixed one to one with Freund´s incomplete adjuvant two weeks after the first injection. An additional injection was given one week later, three weeks after the first injection. Only one blood sample was taken one week after the last injection.

### 4.12. Immunolocalization Studies

Immunostaining was performed, using the following *A. thaliana* specific antibodies, provided by the group of Prof. Chris Franklin in Birmingham. Rat and rabbit anti ASY1 (1:1000, [71]); rat and rabbit anti ZYP1 (1:500, [73]); rat and rabbit anti RAD51 (1:200; [46]) and rabbit anti SPO11-1 (1:100; [46]). The rabbit and mouse antibody against SPO11-2 produced at the Julius Kuehn Institute was used in a 1 to 100 dilution.

## Figures and Tables

**Table 1:** Complementation of *A. thaliana spo11-1-3* and *spo11-2-3* with SPO11-1 or -2 swapped constructs n.d.: not determined.

Construct	Complementing spo11-1-3 Lines	Complementing spo11-2-3 Lines
AthSPO11-1g	6/8	-
Ath SPO11-2g	-	7/8
Ath SPO1swap1	0/19	0/11
Ath SPO1swap2	0/14	0/6
Ath SPO1swap3	0/17	0/8
Ath SPO2swap1	0/11	0/8
Ath SPO2swap2	0/12	0/23
Ath SPO2swap3	0/4	0/9
Ath SPO1swap4	4/21	0/8
Ath SPO2swap4	0/12	0/24
Ath SPO1Δlex	0/30	-
Ath SPO2Δlex	-	0/21

**Table 2:** Pairwise comparison of AthSPO11-1 and AthSPO11-2 to SPO11 proteins from organism used for the complementation approaches.

Organism	Gene	gDNA (bp)	CDS (bp)	Protein (aa)	Ath SPO11-1 (%)	Ath SPO11-2 (%)
*Arabidopsis thaliana*	*SPO11-1*	2633	1089	362	100	*30.5*
	*SPO11-2*	2029	1152	383	*30.5*	100
*Brassica rapa*	*SPO11-1*	2492	1089	362	90.3	30.1
	*SPO11-2*	1870	1143	380	31.8	92.3
*Carica papaya*	*SPO11-1*	4491	1086	361	72.8	32.2
	*SPO11-2*	2163	1149	382	30.5	73.8
*Oryza sativa*	*SPO11-1*	3710	1146	381	58.7	31.4
	*SPO11-2*	2710	1158	385	29.1	62.8

One pair alignment was performed using Lipman Pearson (*K-tuple: 2, Gap penalty 4, Gap length penalty 12).* Highest and lowest identity is shown in bold and the identity between AthSPO11-1 and -2 are shown in italics.

**Table 3:** Complementation of *A. thaliana spo11-1-3* and *spo11-2-3* with SPO11-1 or -2 from different plants.

Line	Construct	Number of Complementing Lines	Average Seed (%) Set Hm-Lines
*spo11-1-3*	BraSPO11-1g	4/4	-
*spo11-2-3*	BraSPO11-2g	11/11	101.4 ± 13.4 (*n* = 18)
*spo11-1-3*	CpaSPO11-1g	0/15	2.49 ± 0.54 (*n* = 15)
*spo11-2-3*	CpaSPO11-2g	0/22	2.6 ± 0.63 (*n* = 19)
*spo11-1-3*	OsaSPO11-1g	0/11	6.47 ±1.34 (*n* = 15)
*spo11-2-3*	OsaSPO11-2g	0/11	3.6 ± 1.36 (*n* = 7)
*spo11-1-3*	AthSPO11-1c	14/17	72.53 ± 12.53 (*n* = 8)
*spo11-2-3-*	AthSPO11-2c	16/16	92.6 ± 11 (*n* = 7)
*spo11-1-3*	BraSPO11-1c	14/14	84.2 ±19.74 (*n* = 6)
*spo11-2-3*	BraSPO11-2c	13/15	100.6 ± 22 (*n* = 7)
*spo11-1-3*	CpaSPO11-1c	3/8	19.04 ± 4.5 (*n* = 11)
*spo11-2-3*	CpaSPO11-2c	0/8	0.72 ± 0.12 (*n* = 13)

Bra = *B. rapa*; Cpa = *C. papaya*; Osa = *O. sativa*; g = genomic DNA; c = cDNA.

## Data Availability

Sequence data from this article can be found in the Arabidopsis Genome Initiative database under the following accession numbers: At3g13170 (Ath SPO11-1), At1g63990 (Ath SPO11-2), At5g20850 (RAD51), Os03g54091 (Osa SPO11-1), Os08g0156900 (Osa SPO11-2), Os03g0284800 (Osa SPO11-4), Gene ID: 5727367 (Cre SPO11), MGI:1349669 (Mmu SPO).

## Supplementary Materials

The following are available online at https://www.mdpi.com/article/10.3390/ijms22179346/s1.
