# Supplementary material for: Heterologous Complementation of SPO11-1 and -2 Depends on the Splicing Pattern
Source: Int J Mol Sci. 2021 Aug 28;22(17):9346. doi: 10.3390/ijms22179346 (PMC8430568; doi:10.3390/ijms22179346)
Supplement: Supplementary file 1 [file ijms-22-09346-s001.zip › ijms-1327431-SM-for publish.pdf]

International Journal of
Molecular Sciences

MDPI

Supplementary Material

Heterologous Complementation of SPO11-1 and -2 Depends on the Splicing Pattern

Thorben Sprink * and Frank Hartung

Julius Kuehn-Institute (JKI)—Federal Research Centre for Cultivated Plants, Institute for Biosafety in Plant Biotechnology, 06484 Quedlinburg, Germany; Frank.Hartung@julius-kuehn.de
* Correspondence: Thorben.Sprink@julius-kuehn.de

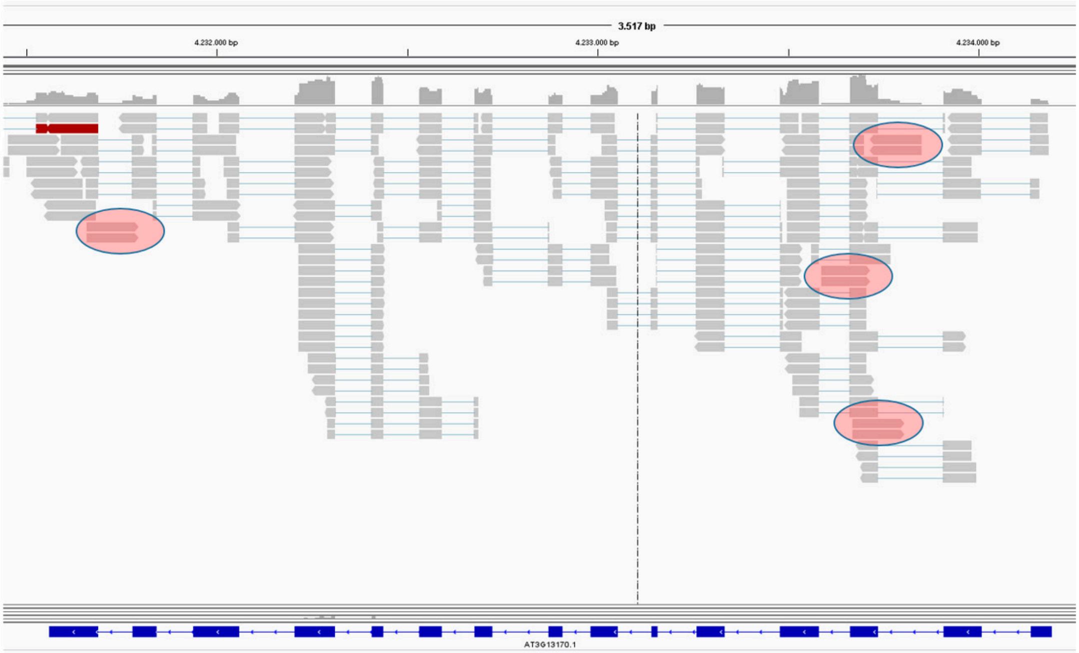

Figure S1. IGV viewer screenshot for SPO11-1 from transcriptome data received from RNA-Seq analysis of Col-0 flower buds. Intron retentions are marked.

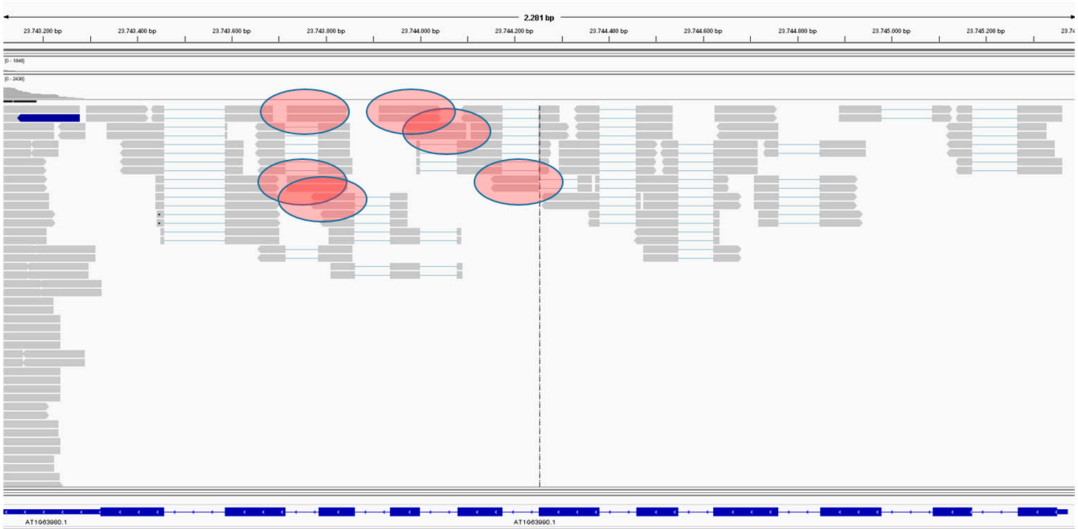

Figure S2. IGV viewer screenshot for SPO11-2 from transcriptome data received from RNA-Seq analysis of Col-0 flower buds. Intron retentions are marked.
